# Spontaneous gas in a retroperitoneal mass: check the testis!

**DOI:** 10.1590/S1677-5538.IBJU.2018.0606

**Published:** 2019-09-02

**Authors:** Jérémy Dana, Florian Maxwell, David. Eiss, Laurence Rocher

**Affiliations:** 1 Department of Diagnostic & Interventional Radiology, Hôpitaux Universitaires Paris Sud, Site Bicêtre, Le Kremlin-Bicêtre, France;; 2 Faculté Paris Sud, Le Kremlin-Bicêtre, France;; 3 IR4M, CNRS, imagerie par résonance magnétique médicale et multi-modalités, CNRS Université Paris Sud, Orsay Cedex, France;; 4 Department of Diagnostic & Interventional Radiology, Hôpital Necker, Paris, France

## Abstract

Testicular germ cell tumor is the most common cancer in 20-to 35-years-old men. There are known risk factors such as undescended testicle(s) and history of testicular cancer. Most lesions are germ cell tumors with two main subtypes: seminomas and non-seminomatous germ cell tumors.

## INTRODUCTION

Testicular germ cell tumor is the most common cancer in 20-to 35-years-old men. There are known risk factors such as undescended testicle(s) and history of testicular cancer. Most lesions are germ cell tumors with two main subtypes: seminomas and non-seminomatous germ cell tumors. Burned out testicular tumor (BOTT) refers to a histological fibrous regression of the primary testicular lesion that generally presents at the stage of metastases (1). This case series shows imaging findings in three men with an atypical presentation of this malignancy, gas-containing retroperitoneal mass revealing in each case a BOTT. To our knowledge, this gas-containing mass has just been illustrated once (2).

## CASE PRESENTATIONS

**Figure 1:**
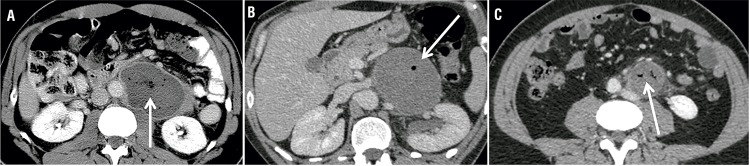
Abdominal contrast-enhanced CT-scan axial cut showing gas-containing retroperitoneal mass (white arrows).

Case 1 - The first patient, a 57-years-old Caucasian man, came to the hospital with afebrile abdominal pain. Physical examination was normal. The biology laboratory results showed slight leukocytosis. Urine culture was sterile. Considering the abdominal pain with leukocytosis, we performed a contrast-enhanced CT-scan. The CT-scan showed a retroperitoneal hypodense mass containing gas (Figure-1A). There was no vascular thrombosis. At first, our diagnostic hypothesis was an abscess. However, there wasn’t any argument for an associated infection such as pyelonephritis, diverticulitis or spondylitis: there was no focal nephritis, bowel fistula or bone abnormalities.

**a)** 57 years old patient; **b)** 41 years old patient; **c)** 34 years old patient; same patient as c) spontaneous decrease in size 4 weeks after the first CT.

**Figure 2:**
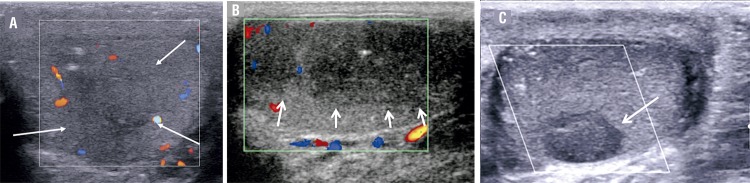
Testicular Color Doppler Ultrasound of three patients (in the same order) demonstrating avascular hypoechoic areas (white arrows) with some microlithiasis suggestive of burned out tumors.

Given a medical history of bilateral undescended testicles treated by orchidopexy in childhood, we carried out a testicular ultrasound (US) showing bilateral testicular atrophy, microlithiasis, and hypoechoic areas involving the left testis (Figure-2A). Nevertheless, the scrotal examination was normal. On the other hand, tumor markers including lactate dehydrogenase, alpha-fetoprotein and human chorionic gonadotropin levels were normal.

The patient underwent radical orchiectomy of the left testis and a seminoma with burned out main component was diagnosed. Finally, the patient underwent a fine needle biopsy of the mass revealing ischemic necrosis without any tumor cell.

Case 2 - The second patient, a 41-years-old Caucasian man, came to the hospital for the same reason, afebrile abdominal pain. Physical examination was also normal. The laboratory results showed slight leukocytosis and urine culture was sterile. In the same way, a contrast-enhanced CT-scan was performed and showed the same results: necrotic retroperitoneal mass containing gas (Figure-1B). The scrotal US revealed a diffuse hypoechoic aspect of the left testis compared to the contralateral testis. Tumors markers were normal.

Radical orchiectomy was performed and pathological examination found hyaline fibrosis without any tumor cell consistent with BOTT (Figure-2B). The CT guided biopsy of the retroperitoneal mass revealed a necrotic seminoma.

Case 3 - The third patient, a 34-years-old Caucasian man came to the hospital with afebrile lumbar pains and presented similar physical examination (including scrotal examination) and laboratory results.

**Figure 3:**
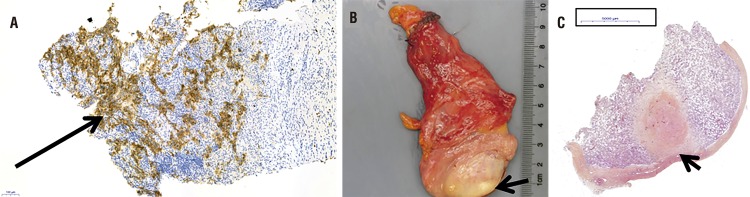
Pathological examinations of the third 34 years old patient.

The contrast-enhanced CT-scan also revealed a gas-containing retroperitoneal mass (Figure 1C). The scrotal US showed a hypoechoic hypovascular area with microlithiasis in the left testis (Figure-2C). Tumor markers were normal. The patient underwent a mass CT guided biopsy and an orchiectomy. The pathological analysis showed respectively necrosis and hyaline fibrosis without tumoral cells. Considering these results, we performed a second biopsy of the retroperitoneal mass that allowed to diagnose lymph node metastasis of a BOTT (Figure-3A). The retroperitoneal mass had spontaneously decreased in size, from 46 to 35mm (largest diameter) during the 4 weeks between the first CT scan and the second biopsy.

**a)** Microscopic view of the second retroperitoneal biopsy using placental alkaline phosphatase (PLAP) marker (arrow), magnifications x 10.9; **b)** Macroscopic view showing a scarred focus (arrow) corresponding to a regressed germ cell tumor; **c)** Microscopic view of the same testis demonstrating prominent lymphoplasmacytic infiltrate around the hyalinized fibrosis (arrow).

To summarize, each patient presented with abdominal pain and leukocytosis revealing gas-containing retroperitoneal mass and hypoechoic hypovascular area with microlithiasis in the testis. In all three cases, tumor markers including lactate dehydrogenase, α-fetoprotein and human chorionic gonadotropin levels were normal.

Pathological examination has been a challenge to diagnose BOTT with metastatic nodes (Figures 3B and 3C) .

After orchiectomy, each patient received chemotherapy by Bleomycin, Etoposide and Cisplatin. They are still free of disease after 14 years (Case-1), 8 years (Case-2) and 1 year (Case-3) of follow-up.

## DISCUSSION

Most reported BOTT are discovered because of symptomatic metastatic nodes, such as presenting with back or flank pain (3-5). Generally, presence of gas in a retroperitoneal mass is usually attributed to retroperitoneal-bowel fistula, abscess or superinfection. BOTT revealed by gas-containing retroperitoneal mass have been shown in only one study to our knowledge (2). This unusual presentation led the authors to perform drainage through endoscopic ultrasound-guided transduodenal puncture. Our cases series reinforces the recommendation of performing scrotal physical exam and scrotal US in case of retroperitoneal masses in men, with aim of avoiding inappropriate management such as extensive surgery or percutaneous drainage. In our series, the presence of gas was attributed to tumor ischemic necrosis. Nevertheless, samples should be sent to culture in order to rule out superimposed infection.

The fibrous replacement and the residual seminomatous part in BOTT may be explained by an intensive immunological response. This mechanism has not been demonstrated in the case of BOTT but an autoimmune response was described in a testicular ‘in situ’ carcinoma (6). Indeed, Lehmann and Müller reported a case in which immunohistochemical examination of the testicular biopsy demonstrated remarkable intracellular and membranous accumulation of IgG antibodies in the atypical spermatogonia. These specific antibodies were found only in the patient’s serum and not in 500 control sera.

The spontaneous regression of the testicular germ cell tumor results in hypoechoic avascular areas corresponding to hyaline fibrosis, and sometimes in atrophy of the testis (7).

Due to the abundance of necrosis, it is sometimes impossible to identify any tumor cell in the retroperitoneal biopsy sample. In these cases, repeat biopsy may be needed. In the second case, the core biopsy was performed with a 16G needle.

Orchiectomy is generally followed by cisplatin-based combination chemotherapy protocols. This therapy is very effective in the treatment of seminomas and non-seminomatous germ cell tumors.

## CONCLUSION

Presence of gas in a retroperitoneal mass is an uncommon presentation of metastatic retroperitoneal lymph nodes of testicular cancer.

It is mandatory to perform a testicular ultrasound in the diagnostic process, despite, sometimes, a normal physical examination. Hypoechoic hypo/avascular areas at US may suggest the diagnosis of BOTT. Retroperitoneal biopsy with a large needle in the less necrotic part may be the key of the diagnosis.
